# Haemosporidian parasites in the ash-breasted Sierra finch (*Geospizopsis plebejus*): insights from an Andean dry forest population

**DOI:** 10.1017/S0031182022001603

**Published:** 2023-01

**Authors:** Xavier Chavarría, Nubia E. Matta, Héctor Cadena-Ortíz, Ibeth Alarcón, Daniela Bahamonde-Vinueza, Angie D. González, Elisa Bonaccorso

**Affiliations:** 1 Laboratorio de Biología Evolutiva, Instituto Biósfera y Colegio de Ciencias Biológicas y Ambientales, Universidad San Francisco de Quito, Quito, Ecuador; 2 Departamento de Biología, Universidad Nacional de Colombia, Sede Bogotá, Colombia; 3 Centro de Investigación de la Biodiversidad y Cambio Climático (BioCamb), Universidad Tecnológica Indoamérica, Machala y Sabanilla, Quito EC170301, Ecuador; 4 Universidad Central del Ecuador, Av. Universitaria, Quito, Ecuador

**Keywords:** Avian malaria, *Babesia*, *Haemoproteus*, morphology, Neotropics, *Plasmodium*

## Abstract

Haemosporidian genera *Plasmodium*, *Haemoproteus* and *Leucocytozoon*, responsible for avian malarial infections, are highly diverse and have a wide range of health effects and predictors, depending on the host and its environmental context. Here, we present, for the first time, detailed information on the identity, prevalence and parasitaemia of haemosporidians and other haemoparasites that infect the ash-breasted Sierra finch, *Geospizopsis plebejus*, in an Andean dry forest. We study the consequences of infection in the host body and health conditions and explore the environmental and intrinsic factors that influence infection status and parasitaemia. We conducted diagnoses by cytochrome b (*cytb*) sequencing and morphological identification, and estimated the levels of parasitaemia based on microscopy. We identified 6 *cytb* lineages infecting *G. plebejus*. Two of them were new lineages: *Haemoproteus* sp. GEPLE01 and GEPLE02. We also detected *Haemoproteus* sp. ZOCAP08, *Haemoproteus* sp. AMAVIR01, *Plasmodium homopolare* BAEBIC02 and *Plasmodium cathemerium* ZONCAP15. By microscopy, we detected *Haemoproteus coatneyi*, *Haemoproteus erythrogravidus*, *P. homopolare* and other unidentified species of *Haemoproteus*, *Plasmodium*, *Babesia* sp. and 1 microfilaria. We found no evidence of *Leucocytozoon*. Additionally, we detected several coinfections by sequencing and microscopy. The prevalence of haemosporidian infections was high (87.7%), and the mean parasitaemia was 61.65 infected cells per 10 000 erythrocytes examined. Prevalence and parasitaemia were higher for *Haemoproteus* than for *Plasmodium*. *Haemoproteus* sp. AMAVIR01 showed the highest prevalence (43.1%) and mean parasitaemia (94.39/10 000 erythrocytes) and might be associated with *H. coatneyi*. Immature individuals showed a lower prevalence than adults, supporting previous findings.

## Graphical abstract

**Figure:**
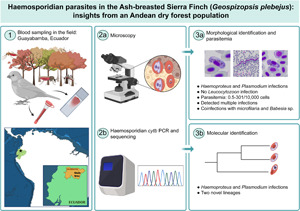

## Introduction

Haemosporidian genera *Plasmodium*, *Haemoproteus* and *Leucocytozoon*, responsible for avian malarial infections, are highly diverse and infect birds in all continents but Antarctica (Valkiūnas, 2005). They are obligate parasites transmitted by haematophagous dipteran vectors that infect blood cells and other host tissues (Valkiūnas, 2005; Santiago-Alarcon *et al*., 2012). The effects of avian malaria may range from mortality (e.g. Atkinson *et al*., 1995; Palinauskas *et al*., 2016) to non-significant reductions in fitness (e.g. Bensch *et al*., 2007; Hammers *et al*., 2016). During the acute, initial phase of the infection, birds may reduce food intake and body weight (Atkinson *et al*., 2000; Valkiūnas *et al*., 2006). They also increase their production of red blood cells (i.e. polychromasia; Palinauskas *et al*., 2022) to counteract the anaemia caused by the parasites (Mitchell and Johns, 2008). Chronic infections are generally associated with milder symptoms (Goswami and Swamy, 2013) but can generate trade-offs between immunological response and reproduction investment (e.g. Nordling *et al*., 1998; Asghar *et al*., 2011), and compromise long-term survival (e.g. Asghar *et al*., 2016).

The course of malarial infection is tightly related to the parasite genus (Atkinson and van Riper, 1991). In general, *Plasmodium* is more pathogenic than *Haemoproteus* (Atkinson and van Riper, 1991; Valkiūnas, 2005), although highly pathogenic species of *Haemoproteus* have been reported (e.g. Sol *et al*., 2003). The pathogenicity of some species of *Leucocytozoon* can be high in domestic poultry and waterfowl, but its impact on other families of birds is poorly known (Atkinson and van Riper, 1991). Also, infections with 2 or more species of haemosporidians are poorly understood (Marzal *et al*., 2008). Still, in a clinical trial comparing single and mixed infections, Palinauskas *et al*. (2018) found that *Plasmodium elongatum* intensity of parasitaemia is enhanced by the presence of *Plasmodium relictum*. Also, in a field-based study, double infections caused a significant decline in body condition compared to single infections (Marzal *et al*., 2008).

Predicting the factors that drive avian malaria epidemiology is challenging because infections respond to a complex interplay between environmental and intrinsic factors. Water availability, in terms of permanent water sources or increased precipitation, tends to predict malaria prevalence (Okanga *et al*., 2013; Ferraguti *et al*., 2018), because it limits vector reproduction and parasite development (Van Riper *et al*., 1986; LaPointe *et al*., 2012). At the same time, host age, sex and body and health conditions can be intrinsic predictors of infection status and intensity of infection. For example, parasite prevalence may decline (e.g. van Oers *et al*., 2010; Hammers *et al*., 2016) or increase (e.g. Cosgrove *et al*., 2008; Knowles *et al*., 2011; Fecchio *et al*., 2015) with age, depending on the host. Also, differences in prevalence between sexes have been related to sexual dimorphism (e.g. Svensson-Coelho *et al*., 2013). The expectation is that males from species with higher dimorphism should suffer from higher prevalence because of the immunosuppressive effect of maintaining secondary sexual characters (Hamilton and Zuk, 1982; Zuk, 1990). However, sex-specific behaviours may cause higher exposure to parasite vectors or generate energy trade-offs between reproduction and immune response (Korpimäki *et al*., 1993; van Oers *et al*., 2010; Baillie *et al*., 2012), increasing the susceptibility of the host. Additionally, weight, body condition or health condition before or during avian malarial infection could exert higher physiological stress on the host and decrease its ability to clear or control the infection (e.g. Lochmiller *et al*., 1993; da Silva Rodrigues *et al*., 2021).

We studied avian malarial infections in a population of the ash-breasted Sierra finch, *Geospizopsis plebejus*. This species is a common dweller of the Andean ecosystems, distributed from Ecuador south to Argentina, along almost the entire elevational range of this mountain chain, from sea level to 4500 m a.s.l. (Campagna *et al*., 2011). It is common in open habitats, including those disturbed by human activities (Jaramillo, 2021). Sexes are dimorphic in plumage (Jaramillo, 2021) but not significantly different in morphological measurements (Llerena-Quiroz, 2018). Females incubate (Hughes, 1980; Pozo-Zamora, 2014), but no additional information on each sex's contributions to reproduction is known. Related species such as plumbeous Sierra finch, *Geospizopsis unicolor* and mourning Sierra finch, *Rhopospina fruticeti*, with similar latitudinal and elevational wide ranges (Campagna *et al*., 2011), have been studied as part of broad haemosporidian surveys (Merino *et al*., 2008; Doussang *et al*., 2021; McNew *et al*., 2021). Still, *G. plebejus* has not received the same level of attention.

Here we present, for the first time, detailed information on the identity, prevalence and parasitaemia of haemosporidians and other haemoparasites that infect *G. plebejus* in an Andean dry forest. We also study the consequences of infection in the host's body and health condition and explore the environmental and intrinsic factors influencing infection status and parasitaemia. Given that, in general, *Plasmodium* tends to be more pathogenic than *Haemoproteus* (Valkiūnas, 2005), we expected to find a higher prevalence and parasitaemia of *Haemoproteus*, because of its reduced fitness cost (Behomme *et al*., 2005). Also, given the potential additive effect of double infections (e.g. Marzal *et al*., 2008; Palinauskas *et al*., 2018), we expected to find higher parasitaemia in individuals with coinfections. We also assumed that body and health conditions would be affected by infection status, parasite genus and coinfections. In terms of predictors, we expected that higher precipitation would favour higher prevalence and parasitaemia, because of increased possibilities for vector development (LaPointe *et al*., 2012). On the other hand, because the effect of age and sex on malarial infections tends to be host-specific, we had no clear expectations about the impact of these 2 variables. Finally, we expected that individuals with poor body and health conditions should have a higher probability of infection and higher parasitaemias than those with good body and health conditions (da Silva Rodrigues *et al*., 2021).

## Materials and methods

### Study area and sampling

We sampled *G. plebejus* at Bosque Protector Jerusalem (BPJ) between December 2012 and June 2013, as part of a broader project focused on generating a community-level baseline for the avian host-parasite dynamics in this site. The BPJ is a public, protected area located in the Guayllabamba valley, 10 km north of Quito, in north-western Ecuador (00°00′17.4″N/078°21′34.7″W; 2000–2500 m a.s.l.). This area encompasses 1110 ha of protected inter-Andean dry forest remnants. It presents seasonality in water availability, with a wet season from October to May (average monthly precipitation for 2012–2013: 66.37 mm) and a dry season from June to September (average monthly precipitation for 2012–2013: 7.7 mm) (data provided by Instituto Nacional de Meteorología e Hidrología, INAMHI). The average temperature remains uniform throughout the year but varies markedly during the day. The average monthly temperature during the study period was 17.3°C, whereas the minimum and maximum average monthly temperatures were 11.8 and 25.2°C, respectively. The vegetation of BPJ is semi-deciduous scrubland of the northern valleys of the Andes (MAE, 2013). Xerophytic species such as Algarrobo (*Acacia macracantha*), yellow trumpetbush (*Tecoma stans*), tuna cactus (*Opuntia soederstromiana*) and quishuar (*Buddleja bullata*) are characteristic of this forest and dominate the landscape (Guerrón *et al*., 2005).

**Fig. 1.**
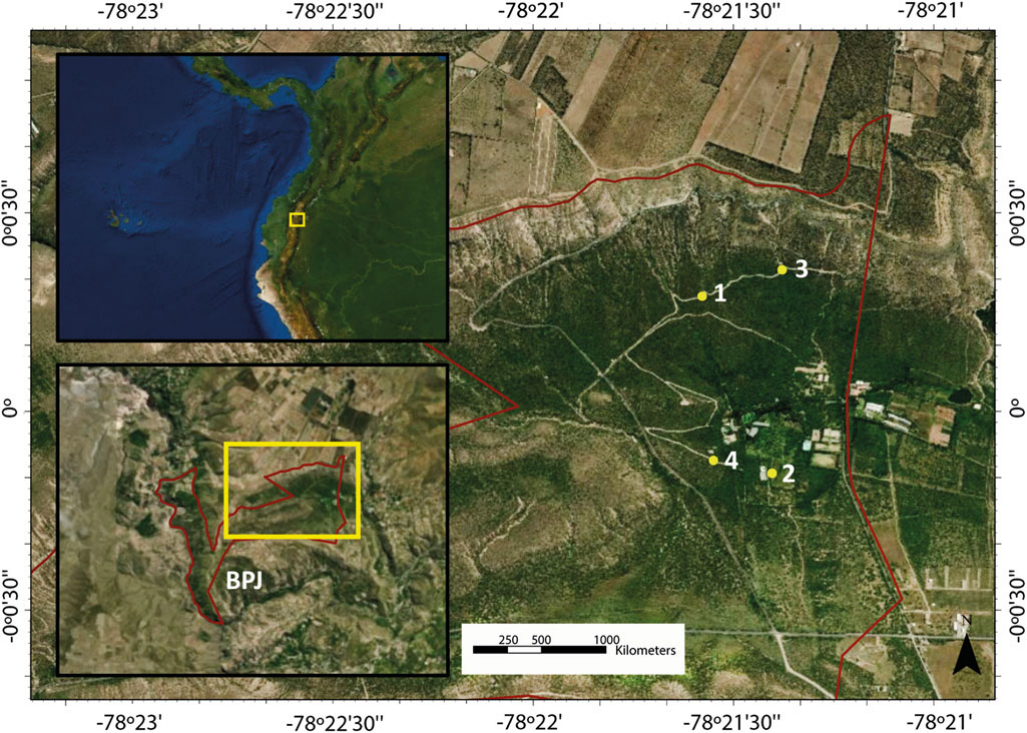
Map of the study area. Sampling sites (1–4) are shown in yellow inside BPJ (red contour).

We set 4 sampling sites encompassing different microclimates, at least 300 m apart (Fig. 1). Sites 1 and 3 were in relatively undisturbed areas along a trail surrounded by native vegetation. Site 2 was located on a plantation of lime (*Citrus* sp.) and avocado (*Persea americana*), and site 4 was in a disturbed area of the forest, used for recreational purposes. Sites 1 and 3 were separated by 600 m from sites 2 and 4. We placed 7 mist nets at each site (dimensions: 12 m × 2.5 m, mesh: 36 mm, 4 shelves), targeting all the species that could get trapped in nets. We sampled each site in 1.5 day monthly visits for a yield of 6 samplings per site, separated by 4 weeks. Each sampling started at 6:00 and ended at 18:00 on the first day and at 12:00 on the second day. All individuals captured were sexed by plumage, measured (tarsus length, bill length, bill width, bill height and wing length) and ringed with plastic bands before release to track recaptures of individuals marked during this study. We drew blood samples from the jugular or brachial veins using syringes attached to 27G disposable needles. Blood smears were prepared in the field using 15–20 *μ*L of blood and then fixed using 100% methanol for 3 min. In the laboratory, fixed blood smears were stained with 10% Giemsa solution for 1 h. The remaining blood samples were stored in 99% ethanol solution for molecular analysis.

### Molecular lineage identification and phylogenetic inference

We extracted whole genomic DNA from blood samples using an in-house protocol (Peñafiel *et al*., 2019). We amplified partial sequences of the mitochondrial cytochrome b (*cytb*) gene of *Plasmodium* and *Haemoproteus* using the HaemF and HaemR2 primers (Bensch *et al*., 2000). Amplification reaction solutions (25 *μ*L volume) consisted of 5 *μ*L of genomic DNA, 1× buffer, 3 mm MgCl_2_, 0.4 mm dNTPs, 0.6 *μ*m HaemF, 0.6 mm HaemR2 and 0.05 U *μ*L^−1^ platinum Taq polymerase (Invitrogen Inc., Carlsbad, USA). We applied a non-nested polymerase chain reaction (PCR) protocol consisting of an initial 3 min denaturation at 94°C; 37 cycles of 94°C denaturation for 30 s, 50°C annealing for 30 s and 72°C extension for 45 s; and 75°C final extension for 10 s. We visualized amplicons by electrophoresis in a 1.2% agarose gel stained with SYBR Safe (Invitrogen Inc., Carlsbad, USA). Positive amplicons were treated with ExoSAP-IT (Affymetrix Inc., Santa Clara, USA) and Sanger sequenced on an ABI 3730XL sequence analyser (Applied Biosystems Inc., Foster City, USA) with the same PCR primers.

We assembled consensus sequences using Geneious 5.1.7 (Kearse *et al*., 2012). All sequences showing no coinfections in chromatograms (i.e. double peaks) were aligned using Clustal X2.1 (Thompson, 1997). We used GenBank and MalAvi BLASTN tools (Zhang *et al*., 2000; Bensch *et al*., 2009) to compare our sequences with previously published ones. New lineages were those with less than 100% query match. We produced separate alignments for unique sequences of *Plasmodium* and *Haemoproteus*, using Clustal X2.1. We used *Leucocytozoon fringillinarum* as the outgroup in both alignments (GenBank accession number: JQ815435). We used Mesquite 3.61 (Maddison and Madison, 2019) to crop the alignment to match the outgroup sequence length (474 base pairs). We also translated the alignment into amino acids to verify the absence of stop codons or indels. Unphased sequences (i.e. showing potential coinfections) were analysed in DnaSP 6.12 (Rozas *et al*., 2017) using the algorithm PHASE (Stephens *et al*., 2001; Stephens and Donnelly, 2003). We ran the program using the FASTA sequences of coinfections with International Union of Pure and Applied Chemistry ambiguity codes, including the unique sequences of the lineages found in the sample. Program settings for the analyses were: 1000 iterations, thinning interval = 10, burn-in = 200 and no recombination.

We used PartitionFinder 2.1.1 to select the best model of molecular evolution for each alignment (Guindon *et al*., 2010; Lanfear *et al*., 2016). The parameters used were branch lengths linked, corrected Akaike information criterion (AICc), and greedy algorithm, an algorithm for heuristic search that increases the efficiency in finding the optimum partitioning scheme while reducing the number of schemes that need to be considered for a given dataset (Lanfear *et al*., 2012). The best partition schemes for *Plasmodium* (by codon position from first to third) were: GTR (generalized time-reversible) + I (invariable sites) + G (gamma distribution); F81 (Felsenstein 4-parameter) and GTR + G, respectively. The best partition schemes for *Haemoproteus* (by codon position from first to third) were: GTR + I + G; GTR + G and GTR + G. We generated phylogenetic trees using MrBayes 3.2.7 for Bayesian inference (Ronquist and Huelsenbeck, 2003) and W-IQ-TREE 1.6.12 for maximum-likelihood inference (Trifinopoulos *et al*., 2016). The analysis in MrBayes was set for 10 million generations, sampling every 1000 trees, discarding 2000 and retaining 8000 trees. The analysis in W-IQ-TREE was set for 10 000 bootstrap replicates using Ultrafast Bootstrap (Hoang *et al*., 2018). The resulting trees were edited with FigTree 1.4.3 (Rambaut, 2017).

### Morphological identification of haemoparasites

Blood smears were double-blind diagnosed and photographed at 1000× magnification using an Olympus BX43 microscope coupled with a DP27 camera and its software, CellSens (Olympus Corporation, Japan). We obtained standard morphometric measurements from uninfected erythrocytes, complex erythrocyte/parasites and parasites using ImageJ software (Schneider *et al*., 2012). Morphological identifications were based on keys from Valkiūnas (2005) and Valkiūnas and Iezhova (2018), as well as recent descriptions of new species (Walther *et al*., 2014; Mantilla *et al*., 2016).

### Prevalence and parasitaemia

We considered a sample as infected if either PCR amplification or microscopic analysis diagnosed it as positive. Parasitaemia values (infected cells per 10 000 erythrocytes) were obtained from Giemsa-stained blood smear counts of positive individuals. Prevalence, mean parasitaemia and confidence intervals were calculated using Quantitative Parasitology 3.0 (Reiczigel *et al*., 2019). Prevalence confidence intervals were calculated using the Sterne method (Reiczigel, 2003), and mean parasitaemia confidence intervals were obtained using the bias-corrected and accelerated (BCa) bootstrap interval method (Rózsa *et al*., 2000). We calculated prevalence and mean parasitaemia for total haemosporidian infections (diagnosed by microscopy or PCR), genus (*Haemoproteus* and *Plasmodium*), parasite lineage and coinfections (detected by sequencing or morphological analysis). We excluded from these analyses all the samples from recaptures of individuals captured during the sampling period.

We compared the prevalence between parasites through chi-square tests, excluding coinfections. Also, after confirming that the parametric assumptions did not hold, we performed unpaired Wilcoxon rank-sum tests to compare the parasitaemia of individuals infected with different genera and those with single infections and coinfections. Finally, we also used Wilcoxon rank-sum tests to compare the body condition (residuals of the regression between body mass and tarsus length) and polychromatophil count per 10 000 erythrocytes examined between individuals infected and non-infected, infected with different parasites and bearing single infections and coinfections. High polychromatophil count, or polychromasia, indicates regenerative anaemia (Jones, 2015), caused by various pathogens and health conditions (Mitchell and Johns, 2008). Thus, it is a measure associated with the health status of the individual (e.g. Travers *et al*., 2010; Schoenle *et al*., 2017). Samples from recaptures were excluded from all analyses.

### Predictors of infection status and parasitaemia

To explore how environmental and intrinsic factors may predict infection status and parasitaemia, we carried out a series of generalized linear model (GLM) analyses in R 1.2.5033 (R Development Core Team, 2013) using the MASS package (Venables and Ripley, 2002). We analysed infection status using a logit model (family = binomial, link = logit), where the response variable was the infection status obtained by PCR or microscopy, and analysed parasitaemia using a negative binomial regression model (link = log), where the response variable was the value of parasitaemia obtained through microscopy. The independent variables (predictors) used in both models were sex, age, body condition, polychromatophil count (as a measure of health condition), sampling site, average relative humidity of the month before the capture day and total precipitation of the month before the capture day. Environmental data were obtained from INAMHI. Totals and averages were calculated from daily values provided by INAMHI.

Model selection was performed as follows. Initially, we obtained a general model that included all factors. Then, we used the dropterm and stepaic functions (MASS package) to obtain 1-term deletions from the original complete model. All models were ranked based on the AICc, using the AICcmodavg package (Mazerolle, 2006) and the chosen (final) model was that with the highest AICc weight. Significance of the final model was assessed in MASS.

## Results

We captured a total of 871 individuals from 36 bird species. Eighty-five birds were *G. plebejus* (9.76% relative abundance) and 65 of those were diagnosed by either molecular or microscopy methods. Out of these 65 individuals, 57 were infected with haemosporidians. Seven individuals were recaptured at least once during the sampling period (8.24%). Out of the recaptured individuals, 2 were captured at site 2 and recaptured at site 4, or vice versa, suggesting at least minimal exchange of individuals between these sites. Details for diagnoses by molecular and microscopic methods are provided in the following sections. Data for all the variables analysed are available in the Supplementary File 1: Dataset S1.

### Molecular identification of haemosporidians

**Fig. 2.**
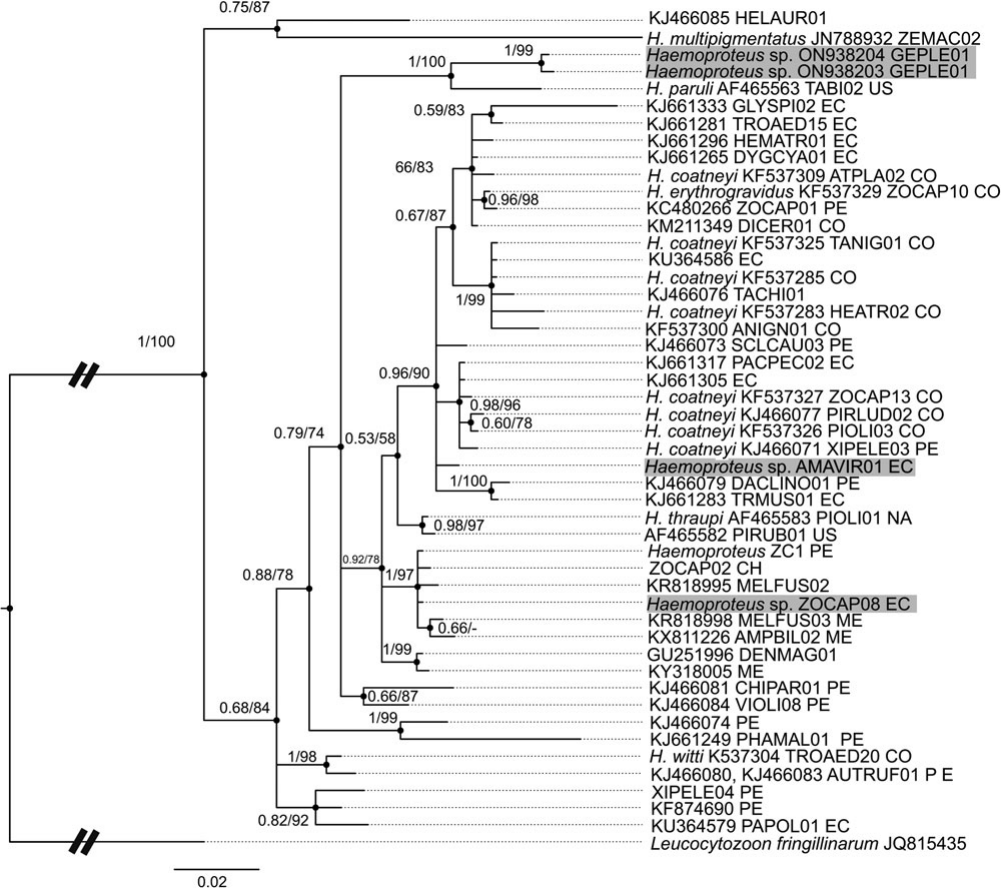
Phylogenetic position of the 4 lineages of *Haemoproteus* (in bold) found in the ash-breasted Sierra finch, *Geospizopsis plebejus*, among related Neotropical lineages. Bayesian posterior probabilities (Bpp) and maximum-likelihood bootstrap supports (MLb) are shown over nodes (Bbb/MLb). Each lineage includes: morphospecies or genus (if available); GenBank accession number (if available); MalAvi name and country where it was detected (CO, Colombia; CH, Chile; EC, Ecuador; ME, Mexico; PE, Peru; US, United States; NA, no information).

**Fig. 3.**
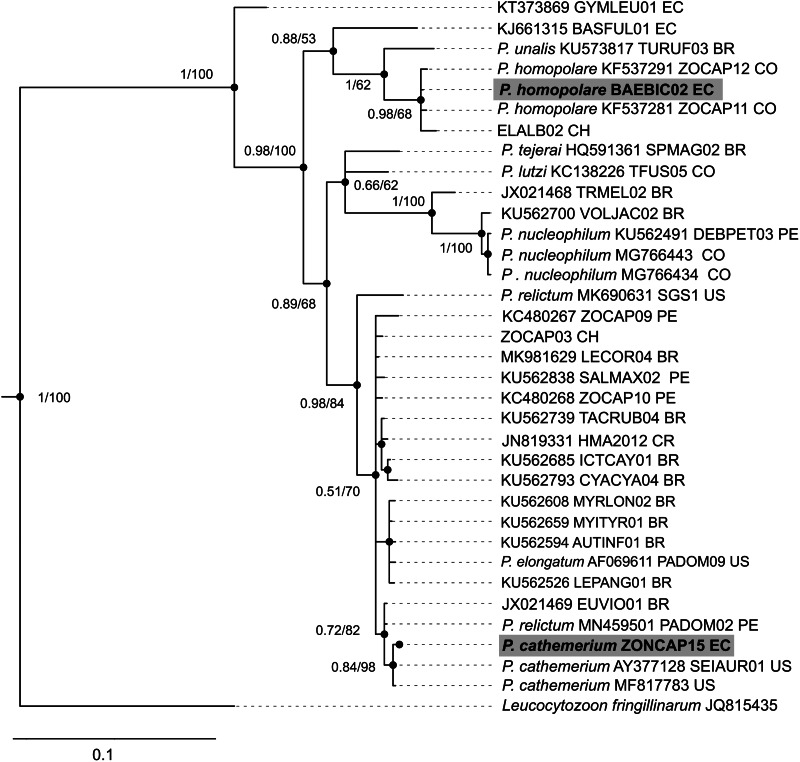
Phylogenetic position of the 2 lineages of *Plasmodium* (in bold) found in the ash-breasted Sierra finch, *G. plebejus*, among related Neotropical lineages. Bpp and MLb are shown over nodes (Bbb/MLb). Each lineage includes: morphospecies or genus (if available); GenBank accession number (if available); MalAvi name (if available) and country where it was detected (BR, Brazil; CO, Colombia; CR, Costa Rica; CH, Chile; EC, Ecuador; PE, Peru; US, United States).

We applied PCR diagnosis to samples of 64 individuals, of which 52 were positive (Table 1). Among these positive samples, we identified 2 new *Haemoproteus* lineages: *Haemoproteus* GEPLE01 and GEPLE02 (GenBank accession numbers: ON938204 and ON938203, respectively). We also found 2 previously reported *Haemoproteus* sp. lineages: ZOCAP08 (originally called ZC1; GenBank: KC480265; Jones *et al*., 2013) and AMAVIR01 (GenBank: JQ988544; McNew *et al*., 2021). However, the phylogenetic position of these 2 lineages was unresolved within the major *Haemoproteus* lineages (Fig. 2). Additionally, samples showed infection by *Plasmodium* (*Haemamoeba*) *cathemerium* ZONCAP15 (GenBank: MK077679; Cadena-Ortiz *et al*., 2019; reported initially as ZOCAP15) and *Plasmodium* (*Novyella*) *homopolare* BAEBIC02 (GenBank: KF537287; González *et al*., 2015). ZONCAP15 grouped with other *P. cathemerium* sequences, whereas BAEBIC02 grouped with *P. homopolare* sequences (Fig. 3).

**Table 1.** Molecular lineages amplified by PCR and morphospecies detected in the ash-breasted Sierra finch, *Geospizopsis plebejus*, at BPJ, Ecuador

Molecular lineage	*n* PCR positive (*n* PCR tested)	Morphospecies by microscopy	*n* positive by microscopy	*n* mic positive (*n* mic tested)
AMAVIR01 (GenBank: JQ988544) *Haemoproteus* sp.	28 (64)	*Haemoproteus coatneyi*	10	27 (65)
		*Haemoproteus erythrogravidus*	7	
		*Haemoproteus* sp.	1	
		Coinfection *H. coatneyi* and *H. erythrogravidus*	1	
		Coinfection *H. coatneyi* and *Haemoproteus* sp.	1	
		Coinfection *H. coatneyi* and *Haemoproteus* sp. 1	1	
		Coinfection *H. coatneyi* and *Haemoproteus* sp. 2	1	
		Coinfection *H. erythrogravidus* and *Haemoproteus* sp. 2	2	
		Coinfection *Haemoproteus* sp., *Haemoproteus* sp. 1	1	
		Coinfection *Haemoproteus* sp. 1, *Haemoproteus* sp. 2	1	
		Coinfection *Haemoproteus* sp., *Babesia* sp.	1	
		1 Negative by microscopy	0	
BAEBIC02 (GenBank: KF537287) *Plasmodium homopolare*	6 (64)	Coinfection *H. erythrogravidus*, *P. (N.) homopolare*	1	6 (65)
		*P. (N.) homopolare*	1	
		Coinfection *Haemoproteus* sp., *P. (N.) homopolare*	2	
		Coinfection *Haemoproteus* sp., *Plasmodium* sp.	1	
		*Plasmodium* sp.	1	
GEPLE01 (GenBank: ON938204) *Haemoproteus* sp.	1 (64)	*Haemoproteus* sp. 2	1	1 (65)
GEPLE02 (GenBank ON938203) *Haemoproteus* sp.	7 (64)	*Haemoproteus* sp.	3	6 (65)
		*Haemoproteus* sp. 3	1	
		Coinfection *Haemoproteus* sp., *Haemoproteus* sp. 2	2	
		1 Negative by microscopy	0	
ZOCAP08 (GenBank: KC480265) *Haemoproteus* sp.	1 (64)	Coinfection *Haemoproteus* sp. 1, *H. erythrogravidus*	1	1 (65)
ZONCAP15 (GenBank: MK077679) *Plasmodium cathemerium*	2 (64)	2 Negative by microscopy	0	0 (65)
Positive but no conting (bad chromatograms)	3 (64)	*H. erythrogravidus*	2	2 (65)
		1 Negative by microscopy		
Negative by PCR, positive by microscopy	7 (64)	*H. coatneyi*	3	7 (65)
		Coinfection *Haemoproteus* sp., *Haemoproteus* sp. 2	1	
		Coinfection *H. coatneyi*, *Haemoproteus* sp. 2 and *microfilaria*	1	
		Coinfection *Haemoproteus* sp., *Plasmodium* sp.	1	
		Coinfection *Haemoproteus* sp., *P. (N) homopolare*	1	
PCR negative	12 (64)			
Microscopy negative				14 (65)
Coinfection infection by sequencing	4 (64)	*H. coatneyi* 2 negative by microscopy	2	2 (65)
Coinfection infection by microscopy				19 (65)

Four samples (HFC-321, HFC-425, HFC-718 and HFC-720) presented double peaks and were phased using DnaSP6 (Supplementary File 1: Coinfections), but only 2-phased sequences produced known lineages. Sample HFC-321 was infected with lineage AMAVIR01 (*Haemoproteus* sp.), which is the most common lineage found to be infecting the species in the study area (see Section ‘Prevalence and parasitaemia’), and a *Haemoproteus* sp. (as determined by BLASTN). Isolate HFC-720 produced lineage BAEBIC02 (*P. homopolare*) and *Plasmodium* sp. (as determined by BLASTN). The lineages in the other 2 isolates (HFC-425 and HFC-718) remained ambiguous; their phased sequences belonged to 2 *Plasmodium* sp. and 2 *Haemoproteus* sp., respectively.

### Morphological identification and its correspondence with molecular lineages

**Fig. 4.**
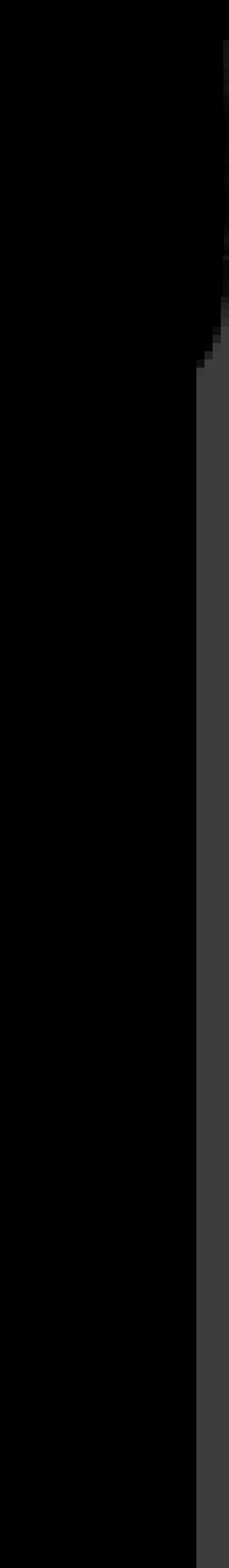
Haemoparasite stages observed in the ash-breasted Sierra finch, *G. plebejus*. (A) Microgametocyte, and (B) macrogametocyte of *Haemoproteus coatneyi*, (C) microgametocyte, and (D) macrogametocyte of *Haemoproteus erythrogravidus*, (E) erythrocytic meronts, (F) gametocyte of *Plasmodium homopolare* and (G, H) erythrocytic meronts of *Babesia* sp. Scale bar = 10 *μ*m. (I, J) Confections of *Plasmodium* and *Haemoproteus*. Scale bar = 10 *μ*m. (K, L) Microfilaria. Scale bar = 20 *μ*m. Black arrowheads: pigment granules, double black arrowheads: merozoites. White arrowheads, protrusions of the erythrocyte membrane as the most relevant characteristics of *H*. *erythrogravidus*. Giemsa-stained thin blood smears. (A–H) At high magnification 1000×; (I–L) at low magnification 400×.

We performed morphological identification on 65 individuals (Table 1), of which 51 were positive. Identified haemosporidians were as follows: *Haemoproteus coatneyi*, *Haemoproteus erythrogravidus*, *P. homopolare* (Fig. 4), 1 *Plasmodium* sp. and 3 new potential species of *Haemoproteus*. Additionally, we observed a coinfection of *Haemoproteus* sp. with *Babesia* sp. (isolate HFC-505) and another of *H. coatneyi*, *Haemoproteus* sp. and microfilaria (isolate HFC-677) (Fig. 4). We found no evidence of infection by *Leucocytozoon*.

The correspondence between molecular lineages and morphospecies of haemosporidians is presented in Table 1. Seven out of 8 samples carrying lineages GEPLE01 or GEPLE02 were infected with different species of *Haemoproteus*. Out of 28 samples carrying lineage AMAVIR01, 14 were infected with *H. coatneyi* in single infections (10 samples) or coinfections (4 samples); the remaining samples showed *H. erythrogravidus* in single infection (7 samples) or coinfections (2 samples), or *Haemoproteus* sp. in single infections or coinfections. The only sample carrying lineage ZOCAP08 was associated with coinfection by *H. erythrogravidus* and an unidentified *Haemoproteus* species (*Haemoproteus* sp. 1). In the 6 samples carrying BAEBIC02, we identified *P. homopolare* and *Plasmodium* sp. in single infections or with *H. erythrogravidus*, and an unidentified *Haemoproteus* species. Only the molecular diagnosis detected infections by ZONCAP15. Also, we found mismatches between molecular and morphological diagnoses of coinfections (Table 1). None of the 19 coinfections detected by microscopy was detected by sequencing. Also, among the 4 samples with coinfections detected by sequencing, 2 were diagnosed as single infections of *H. coatneyi* by microscopy, and 2 had negative diagnoses.

Finally, we obtained diagnoses of the recapture(s) of 7 individuals (Table 2). Time lapses from capture to first or second recapture varied between <1 and 4 months. Unfortunately, the first capture of 1 individual (610 R), negative by microscopic diagnosis, could not be molecularly diagnosed. All the other individuals were infected in their first capture and in subsequent recaptures. Five of those were infected with the same lineage within <1 and 2 months.

**Table 2.** Molecular and morphological diagnosis of haemosporidian parasites in individuals of the ash-breasted Sierra finch, *G. plebejus*, captured and recaptured at BPJ, Ecuador

Individuals with multiple captures	Sample code	Date of capture^a^	Site	Molecular lineage	Morphological diagnosis
538 R	HFC-045	2012-12-03	1	AMAVIR01	*H. coatneyi*
	HFC-244	2013-01-11	1	AMAVIR01	*H. coatneyi*
610 R	HFC-165	2012-12-27	4	BAEBIC02	*Haemoproteus* sp., *Plasmodium* sp.
	HFC-341	2013-01-26	4	BAEBIC02	*P. homopolare*, *Haemoproteus* sp.
	HFC-748	2013-04-27	4	Positive, no contig obtained	*H. erythrogravidus*, *P. homopolare*
616 R	HFC-194	2012-12-28	4	No PCR performed	Negative
	HFC-421	2013-02-09	2	BAEBIC02	*Plasmodium* sp.
	HFC-495	2013-02-23	4	BAEBIC02	Negative
661 R	HFC-271	2013-01-13	2	BAEBIC02	*H. erythrogravidus*, *Plasmodium* sp.
	HFC-356	2013-01-26	4	BAEBIC02	*H. erythrogravidus*, *P. homopolare*
677 R	HFC-297	2013-01-19	3	GEPLE02	*Haemoproteus* sp.
	HFC-718	2013-04-20	3	Multiple infection, ambiguous	Negative
682 R	HFC-019	2012-12-02	1	GEPLE02	*Haemoproteus* sp., *Haemoproteus* sp. 2
	HFC-386	2013-02-02	1	GEPLE02	Negative
1071 R	HFC-610	2013-03-29	3	ZONCAP15	Negative
	HFC-720	2013-04-20	3	BAEBIC02, *Plasmodium* sp.	*H. coatneyi*

a Year, month, day.

### Prevalence and parasitaemia

We found a high prevalence of haemosporidian parasite infections, with 87.7% of infected individuals (57 infected/65 analysed). Mean parasitaemia was 61.65 infected cells per 10 000 cells (*N* = 57) (Table 3). The prevalence of *Haemoproteus* molecular lineages was 56.9%, and their mean parasitaemia was 82.14 infected cells per 10 000 cells. The prevalence of *Plasmodium* lineages was 12.3%, and their mean parasitaemia was 22 infected cells per 10 000 cells. Prevalence and parasitaemia were higher for *Haemoproteus* lineages than for *Plasmodium* lineages (prevalence: chi-square = 28.58, *df* = 1, *P* < 0.0001; parasitaemia: *W* = 225, *P* = 0.023). The *Haemoproteus* sp. AMAVIR01 lineage showed the highest prevalence (43.1%) and highest mean parasitaemia (mean = 94.39 infected cells per 10 000 cells). The highest parasitaemia was found in HFC-695 sample (301 infected cells per 10 000 cells), which carried the AMAVIR01 lineage and *H. erythrogravidus* morphology. The only individual (HFC-535) infected with lineage ZOCAP08 (*Haemoproteus* sp.) presented high parasitaemia, with 219 infected cells per 10 000 cells. Microscopy of this sample revealed a coinfection between *H. erythrogravidus* and *Haemoproteus* sp. The prevalence of *Babesia* sp. and microfilariae was 1 in 65 individuals (1.54%).

**Table 3.** Prevalence and mean parasitaemia by haemosporidian parasites per parasite species and lineage the ash-breasted Sierra finch, *G. plebejus*, at BPJ, Ecuador

Dominant *cytb* lineage in the sample and number of positive samples	Prevalence^a^ (*n* = 65)	Prevalence 95% CI^b^	Prevalence 99% CI	Mean parasitaemia or parasitaemia (when *N* = 1)^c^	Mean parasitaemia 95% CI	Mean parasitaemia 99% CI
Haemosporida (either *Haemoproteus* or *Plasmodium* infections^d^) (*n* = 57)	0.877	0.7711–0.9422	0.7360–0.9538	61.65	46.42–84.63	42.49–91.25^e^
*Haemoproteus* sp. all molecular lineages (*n* = 37)	0.569	0.4459–0.6855	0.4057–0.7198	82.14	60.24–112.54	54.97–127.76^e^
*Plasmodium* sp. all molecular lineages (*n* = 8)	0.123	0.0578–0.2289	0.0462–0.2640	22	7.00–58.13^e^	5.38–66.38^e^
*Haemoproteus* sp. AMAVIR01 (*n* = 28)	0.431	0.3145–0.5541	0.2802–0.5943	94.39	69.25–131.96	63.25–144.61^e^
*Haemoproteus* sp. GEPLE01 (*n* = 1)	0.015	0.0008–0.0820	0.0002–0.1071	21	–	–
*Haemoproteus* sp. GEPLE02 (*n* = 7)	0.108	0.0517–0.2062	0.0370–0.2409	22.29	6.00–53.86	3.14–60.00^e^
*Haemoproteus* sp. ZC1 ZOCAP08 (*n* = 1)	0.015	0.0008–0.0820	0.0002–0.1071	219	–	–
*P. cathemerium* ZONCAP15 (*n* = 2)	0.031	0.0055–0.1054	0.0024–0.1376	0.50	0.00–0.50^e^	0.00–1.00^e^
*P. homopolare* BAEBIC02 (*n* = 6)	0.092	0.0410–0.1903	0.0282–0.2246	29.17	10.50–66.83	7.50–83.17^e^
Multiple infections by sequencing (*n* = 2)	0.031	0.0055–0.1054	0.0024–0.1376	5.50	0.00–5.5^e^	0.00–11.00^e^
Multiple infections by microscopy (*n* = 19)	0.292	0.1904–0.4149	0.1615–0.4528	82.42	56.89–124.16	51.68–144.84^e^
Lineage undetermined (*n* = 3)	0.046	0.0127–0.1285	0.0068–0.1614	6	0.00–8.67^e^	0.00–9.33^e^

– denotes that no confidence interval is presented because of *n* = 1.

a Prevalence was calculated taking into account all positive samples by molecular and microscopic analysis excluding samples of recaptured individuals.

b Confidence interval. Prevalence confidence intervals were calculated using Sterne's method. Mean parasitaemia confidence intervals were calculated using the BCa bootstrap interval method.

c Parasitaemia was calculated from positive PCR samples, through microscopy (number of infected erythrocytes in 10 000 cells counted).

d Detected by molecular diagnosis or microscopy.

e Uncertain confidence intervals because of a small number of replicates.

We found no differences in body condition or polychromatophil count between positive and negative samples (body condition: *W* = 140, *P* = 0.13; polychromatophil count: *W* = 230, *P* = 0.98), samples carrying *Haemoproteus* and *Plasmodium* lineages (body condition: *W* = 142, *P* = 0.76; polychromatophil count: *W* = 127, *P* = 0.54), or samples carrying single infections and single coinfections (body condition: *W* = 276, *P* = 0.33; polychromatophil count: *W* = 368.5, *P* = 0.19). Parasitaemia was also not significantly different between samples carrying single infections and coinfections (*W* = 263.5; *P* = 0.059), but marginally significant results suggest that parasitaemia might be higher for coinfections if a larger sample was available.

### Predictors of infection status and parasitaemia

In the analysis of predictors of infection status, model selection by AICc of the logit models retained a model with host age as the only predictor for infection status (AICcWt = 0.50; Table 4). According to this model, immature individuals show a lower prevalence than adults (Table 5). For parasitaemia, model selection by AICc of the negative binomial models retained the null model as the best model (AICcWt = 0.62; Table 6). This result precluded further exploration of the predictors of parasitaemia.

**Table 4.** Model selection criteria for the predictors of infection status by haemosporidian parasites (logit model) in individuals of the ash-breasted Sierra finch, *G. plebejus*, at BPJ, Ecuador

Model	Predictors	*K*	AICc	Delta AICc	AICc weight	Cum. weight	LL
Model 7	Age	2	43.95	0.00	0.50	0.50	−19.86
Model 6	Age + precipitation	3	45.30	1.34	0.26	0.76	−19.42
Model 5	Age + precipitation + polychromatophil count	4	46.83	2.88	0.12	0.88	−19.02
Null model	Null model	1	48.01	4.06	0.07	0.94	−22.97
Model 4	Age + precipitation + humidity + polychromatophil count	5	48.95	5.00	0.04	0.98	−18.88
Model 3	Age + precipitation + sex + humidity + polychromatophil count	6	51.45	7.49	0.01	1.00	−18.87
Model 2	Age + precipitation + sex + body condition + humidity + polychromatophil count	7	54.04	10.09	0.00	1.00	−18.86
Model 1	Age + precipitation + site + sex + body condition + humidity + polychromatophil count	10	58.84	14.79	0.00	1.00	−16.93

AICc, corrected Akaike information criterion; Cum., cumulative.

**Table 5.** Coefficient summary of the model for infection status by haemosporidian parasites (logit model) in individuals of the ash-breasted Sierra finch, *G. plebejus*, at BPJ, Ecuador

Model	Response	Predictors	Estimate	Std. error	*Z*-value	*P* value
Model 6	Infection status	Intercept	2.37	0.52	4.54	<0.001
		Age (immature)	−2.15	0.85	−2.53	0.011

**Table 6.** Model selection criteria for the predictors of parasitaemia by haemosporidian parasites (negative binomial model) in the ash-breasted Sierra finch, *G. plebejus*, at BPJ, Ecuador

Model	Predictors	*K*	AICc	Delta AICc	AICc weight	Cum. weight	LL
Null model	Null model	2	449.06	0.00	0.62	0.62	−222.38
Model 5	Polychromatophil count + site + body condition	7	451.31	2.25	0.20	0.82	−217.01
Model 4	Humidity + polychromatophil count + site + body condition	8	452.14	3.08	0.13	0.95	−215.89
Model 3	Humidity + polychromatophil count + site + body condition + sex	9	454.59	5.53	0.04	0.99	−215.48
Model 2	Humidity + polychromatophil count + site + body condition + precipitation + sex	10	457.23	8.17	0.01	1.00	−215.07
Model 1	Humidity + polychromatophil count + site + body condition + precipitation + age + sex	11	460.83	11.77	0.00	1.00	−215.01

AICc, corrected Akaike information criterion; Cum., cumulative.

## Discussion

### Molecular and morphological diagnosis of haemosporidian parasites

We identified 6 *cytb* haemosporidian lineages infecting *G. plebejus*: *Haemoproteus* sp. GEPLE01 and GEPLE02, *Haemoproteus* sp. AMAVIR01, *Haemoproteus* sp. ZOCAP08, *P. homopolare* BAEBIC02 and *P. cathemerium* ZONCAP15. Lineages GEPLE01 and GEPLE02 are novel and are the 4th and 5th haemosporidian lineages reported for this species, after lineages *Leucocytozoon* ANIIGN02 and *Haemoproteus* CONCIN03 from Peru (McNew *et al*., 2021), and PHRPLE01 from Chile (Doussang *et al*., 2021). Our results suggest that GEPLE01 or GEPLE02 originated as a single, silent mutation of the other. Thus, differences in prevalence and mean parasitaemia are unrelated to their molecular identity for the *cytb* fragment analysed herein. According to the morphological data, these lineages could be associated with the unidentified *Haemoproteus* sp. 2 or *Haemoproteus* sp. 3 morphologies (Table 1). In-depth analyses must be performed to determine if they correspond to new species.

The most common lineage in this study was *Haemoproteus* sp. AMAVIR01. The only known host infected by *Haemoproteus* sp. AMAVIR01 is the hummingbird *Amazilia viridicauda* (GenBank: JQ988544) from Calca (Peru) at an elevation of 2953 m a.s.l. (McNew *et al*., 2021; Dataset_S01). To date, no morphological identification has been provided for this lineage. According to our results, it is most likely associated with morphospecies *H. coatneyi* (Table 1). The capacity to infect Apodiformes and Passeriformes may indicate that this lineage is host-generalist, which is not common but has been reported for *Haemoproteus* (e.g. Moens *et al*., 2016). An alternate explanation is that since this lineage was detected in *A. viridicauda* only by sequencing (no microscopy was applied), this hummingbird might not be a competent host for *Haemoproteus* sp. AMAVIR01. A positive PCR diagnosis might result from sporozoite-stage infection and abortive development in the hosts (Valkiūnas *et al*., 2014). This possibility highlights the importance of combining microscopy and molecular diagnosis when studying avian haemosporidian parasites (Palinauskas *et al*., 2016).

The lineage *Haemoproteus* sp. ZOCAP08 and both *Plasmodium* lineages, ZONCAP15 and BAEBIC02, infect the rufous-collared sparrow, *Zonotrichia capensis*, in the study area (Cadena-Ortiz *et al*., 2019). Lineage ZOCAP08 infects at least 12 other avian species from North America to Argentina (Jones *et al*., 2013; Reinoso-Pérez *et al*., 2016; Carbó-Ramírez *et al*., 2017; Ham-Dueñas *et al*., 2017; Fecchio *et al*., 2019; Barrow *et al*., 2021; McNew *et al*., 2021) but has not been assigned a morphospecies in the MalAvi repository. To date, it has been attributed to *H. coatneyi* (González *et al*., 2015) and *Haemoproteus* sp. (Carbó-Ramírez *et al*., 2017). Our study suggests an association with *H. erythrogravidus* and *Haemoproteus* sp. 1., but this result comes from a single positive sample that could have been infected by multiple morphospecies. Thus, more research is needed to support an association between *Haemoproteus* sp. ZOCAP08 and a specific morphology. *Plasmodium cathemerium* ZONCAP15 was found to be infecting 2 individuals with low parasitaemia. This lineage also showed low prevalence (2.26% by molecular diagnosis) in *Z. capensis* analysed for the same period and by the same methodology (Cadena-Ortiz *et al*., 2019). However, the only sample diagnosed by microscopy showed one of the highest parasitaemias found for that host (Cadena-Ortiz *et al*., 2019). *Plasmodium cathemerium* is a well-known generalist that infects many hosts in different avian taxa and is highly pathogenic (Vanstreels *et al*., 2015), which could explain its low prevalence. Still, monitoring this parasite in other host species is needed to understand its prevalence and pathogenicity. Finally, lineage BAEBIC02 *P. homopolare* is widespread in the Americas, from Alaska to Peru, and has been found in at least 15 other avian species (Martinsen *et al*., 2007; Galen and Witt, 2014; Oakgrove *et al*., 2014; Walther *et al*., 2014; González *et al*., 2015; Marzal *et al*., 2015). Finally, our recapture data, showing persistent haemosporidian infections, are valuable because of the lack of repetitive measures in field studies. However, a higher sample and serial recaptures would be necessary to analyse changes in host condition and parasitaemia during the course of the infection.

It is important to state that our study may be underestimating the diversity of haemosporidians in *G. plebejus* because we did not use the nested PCR approach of Hellgren *et al*. (2004), which is the standard for current avian malaria studies. In their methodology, a fragment of haemosporidian *cytb* is amplified, and then a second PCR is applied on that fragment to amplify either *Leucocytozoon*, or *Plasmodium* and *Haemoproteus*. Here, we used only the second step for amplifying *Plasmodium* and *Haemoproteus*, as originally proposed by Bensch *et al*. (2000). Failing to use the nested PCR approach may have decreased the sensibility of the PCR assay in detecting several infections, especially low-intensity *Plasmodium* sp. infections (Waldenström *et al*., 2004). This methodological approach may also be responsible for the small number of coinfections detected by PCR and at least some discrepancies between the molecular and microscopic diagnosis in detecting coinfections.

### Morphological diagnosis of other haemoparasites

We found no evidence of *Leucocytozoon* infection. Based on the distribution of blackflies (Simuliidae), Lotta *et al*. (2016) suggested that the transmission of this parasite is optimal above 2400 m a.s.l. but it may start as low as 2000 m a.s.l. This elevational limit seems to be related to the environmental conditions required by the life cycle of its vectors, blackflies (Simuliidae), in the Andes (Matta *et al*., 2014). Our sample site lies between 2000 and 2500 m a.s.l., and 2 blackfly species occur in the area, 1 of them at a relatively high abundance (Subía-Solís, 2013). Although we found no evidence of *Leucocytozoon* infection by microscopy, this parasite might persist at low parasitaemia and remain undetected. Thus, PCR amplification of the parasite DNA in avian hosts and blackflies of this community would be advisable in future studies.

We detected a microfilaria in 1 sample out of 65 samples diagnosed by microscopy (1.5% prevalence). The prevalence of microfilaria infection in Andean birds at the community level is relatively low, in the order of 0‒3% (2.3%, Bennett and Borrero, 1976; 3%, Valkiūnas *et al*., 2003; 0%, Munro *et al*., 2009; 2.9%, Rodríguez *et al*., 2009). However, even if they show low prevalence, diagnosing and reporting microfilariae is important since they may interact with malarial infections, affecting the host's health condition (Clark *et al*., 2016).

We also found 1 sample infected with *Babesia* (Apicomplexa: order Piroplasmida). Parasites in this genus are transmitted by ticks (order Ixodida) and are known to have zoonotic potential (Yabsley and Shock, 2013; Shock *et al*., 2014). *Babesia* species have been detected in several bird families, including Passeriformes and non-Passeriformes (Peirce, 2000; Yabsley *et al*., 2017; Ebani and Mancianti, 2021). However, instances of infections in Neotropical birds have been poorly documented. There are reports of infection in Orinoco geese, *Neochen jubata*, in Brazil (Werther *et al*., 2017), and veery, *Catharus fuscescens*, in Canada (Scott *et al*., 2019). To our knowledge, the only known Passerines captured in South America with a *Babesia* sp. infection are the Juan Fernández turdus, *Turdus falcklandii*, and Juan Fernández tit-tirant, *Anairetes fernandezianus*, from Juan Fernández archipelago, and *T. falcklandii* from mainland Chile (Martínez *et al*., 2015). Woodworth-Lynas *et al*. (1989) reported *Babesia* in Brazil, but collectively, as part of ‘other’ parasitic genera; therefore, no specific information was provided. Thus, much research is needed to understand *Babesia* parasites in Passeriformes, especially in South America and the Neotropics.

### Patterns of prevalence and parasitaemia

Molecular diagnosis revealed a high prevalence of haemosporidian infections in the population. This result can be related to the high abundance of the host in the study area, the 3rd most abundant in the community. The 1st and 2nd most abundant species are the *Z. capensis* and the common ground dove, *Columbina passerina*, which also show high haemosporidian prevalence and parasitaemia (Cadena-Ortiz *et al*., 2019; DB, HFC and EB, unpublished data). Considering the relative abundance of the host is important because prevalence usually increases alongside local host abundance (e.g. Ricklefs *et al*., 2005; Matthews *et al*., 2016). Still, a more comprehensive sampling of the avian community at this site is necessary to determine if this pattern holds.

*Haemoproteus* spp. were 4 times more prevalent than *Plasmodium* spp., which is consistent with previous studies (Bensch *et al*., 2000; Clark *et al*., 2014), but the prevalence of *Plasmodium* could be particularly underestimated because of our choice of a non-nested PCR approach. However, even if we failed to detect several *Plasmodium* infections in the sample, the morphological identification also detected a low prevalence of *Plasmodium*. This result suggests that high-intensity infections by *Plasmodium* are rare or that affected individuals reduce their activity, lowering their capture probability.

Our results point to higher parasitaemia by *Haemoproteus* than *Plasmodium*, which is also consistent with previous studies (e.g. Fallon and Ricklefs, 2008; Rodríguez-Hernández *et al*., 2021). Contrary to our expectations, we found no effect of infection, lineage or coinfection on body condition or polychromatophil count. Lack of differences in body condition have been observed between infected and non-infected individuals (Granthon and Williams, 2017), and, in the form of body weight, between single infections and coinfections with 2 different species and lineages of *Plasmodium* (Palinauskas *et al*., 2018, 2022). We expected that polychromatophil count would differ, at least between infected and non-infected individuals. However, since polychromasia increases with parasitaemia (Palinauskas *et al*., 2022), an individual with low parasitaemia may have similar levels of polychromasia as a non-infected one. Still, uncovering some of these relationships may be hampered by our methodological limitations in PCR diagnosis and relatively small sample size.

On the other hand, we found that parasitaemia was marginally higher in samples with coinfections than in samples with single infections. This trend coincides with the results of Palinauskas *et al*. (2018) when simulating coinfections by 2 different species of *Plasmodium*. However, to understand the underlying processes that regulate parasitaemia of coinfections, more studies are needed on the interactions between different species and lineages of haemosporidians, and among them and the immune system of the host.

### Predictors of prevalence and parasitaemia

The age of the host was the only predictor of infection status, with immature ones showing lower prevalence (63%) than adults (97%). These results are coherent with previous studies in Neotropical and temperate birds (e.g. Wood *et al*., 2007; Fecchio *et al*., 2015; Cadena-Ortiz *et al*., 2019). Prevalence may increase with age as survivors acquire immunity to the parasite (see Atkinson *et al*., 2001). However, the relationship between age and prevalence must be context-dependent, in terms of both the environment and natural history of the host, since other studies have found that prevalence increases with age (e.g. van Oers *et al*., 2010; Hammers *et al*., 2016).

Parasitaemia, on the other hand, was not predicted by any of the variables included in the GLMs. Negative results for all but 1 predictor of prevalence and all predictors of parasitaemia might result from our relatively small sampling effort because of 2 main reasons. First, although our sampling size might be adequate for other species, it might not be for species with a high prevalence of infection. When non-infected individuals are rare, parameter value estimation for these individuals is challenging. Second, parasitaemia variance is naturally high, which may also complicate parameter estimation with relatively reduced sample sizes. Still, some additional factors should be considered to better understand this system. On the other hand, we found no effect of precipitation on prevalence and parasitaemia, which was surprising, considering that there was variation in total precipitation in the month before the capture day, from 16.7 to 91.8 mm, more than a 5-fold difference. Cadena-Ortiz *et al*. (2019) found an effect of precipitation on haemosporidian prevalence for *Z. capensis* in the same area, using the same sampling and parasite-screening methodology. This difference might be explained by host ecology, physiology and differences among infecting parasites, or, again, a higher sampling size (i.e. 177 individuals).

We also found no effect of host sex on prevalence or parasitaemia, which might result from an interplay of several factors. Males of *G. plebejus* may maintain secondary sexual characters at an immunological cost (Hamilton and Zuk, 1982; Zuk, 1990), although this species' dimorphism is moderate (see plates in Jaramillo, 2021). On the other hand, females may pay a similar or higher cost by exposing themselves to vector bites during incubation. Still, more information on the reproductive behaviour of males and females of this species is necessary to posit and test more informed hypotheses.

Finally, other factors not measured herein might predict prevalence and parasitaemia better. First, given the seasonality of the study area, a year-long analysis is necessary to determine more adequately the relationship between malarial infection and environmental factors. Second, other variables such as the abundance of and distance to water sources, vegetation structure and remote-sensing derived data (e.g. normalized difference vegetation index) may help documenting the heterogeneity among sites (e.g. Hernández-Lara *et al*., 2017; Ferraguti *et al*., 2018). Also, additional measures of health condition (e.g. heterophil/lymphocyte ratio, haematocrit level), and individuals' reproductive status may generate more robust predictions of both prevalence and parasitaemia.

## Data Availability

Unique DNA sequences for haemosporidian parasites were uploaded to GenBank under accession nos. ON938203 and ON938204 (https://www.ncbi.nlm.nih.gov/genbank/). Raw data are available in Supplementary File 1.

## Supplementary material

For supplementary material accompanying this paper visit https://doi.org/10.1017/S0031182022001603. click here to view supplementary material
